# Discussion on a Vehicle–Bridge Interaction System Identification in a Field Test

**DOI:** 10.3390/s23010539

**Published:** 2023-01-03

**Authors:** Ryota Shin, Yukihiko Okada, Kyosuke Yamamoto

**Affiliations:** 1 Graduate School of Systems and Information Engineering, University of Tsukuba, 1-1-1 Tennodai, Tsukuba 305-8573, Ibaraki, Japan; 2 Institute of Systems and Information Engineering, University of Tsukuba, 1-1-1 Tennodai, Tsukuba 305-8573, Ibaraki, Japan; 3 Center for Artificial Intelligence Research, University of Tsukuba, 1-1-1 Tennodai, Tsukuba 305-8577, Ibaraki, Japan

**Keywords:** drive-by bridge monitoring, vehicle–bridge interaction, system identification, field test

## Abstract

For infrastructures to be sustainable, it is essential to improve maintenance and management efficiency. Vibration-based monitoring methods are being investigated to improve the efficiency of infrastructure maintenance and management. In this paper, signals from acceleration sensors attached to vehicles traveling on bridges are processed. Methods have been proposed to individually estimate the modal parameters of bridges and road unevenness from vehicle vibrations. This study proposes a method to simultaneously estimate the mechanical parameters of the vehicle, bridge, and road unevenness with only a few constraints. Numerical validation examined the effect of introducing the Kalman filter on the accuracy of estimating the mechanical parameters of vehicles and bridges. In field tests, vehicle vibration, bridge vibration, and road unevenness were measured and verified, respectively. The road surface irregularities estimated by the proposed method were compared with the measured values, which were somewhat smaller than the measured values. Future studies are needed to improve the efficiency of vehicle vibration preprocessing and optimization methods and to establish a methodology for evaluating accuracy.

## 1. Introduction

Transportation and logistics are supported by civil structures. Civil structures deteriorate due to daily use and natural disasters. If the deterioration of infrastructures is left unattended, it will lead to structural damage. Structural damage sometimes causes serious accidents, with economic consequences and loss of life. Therefore, infrastructure development is essential, and structure health monitoring (SHM) is attracting attention in many countries [1,2,3]. Infrastructures are maintained and managed through detailed inspections, but inspection costs are high. Therefore, methods focusing on vibration have been proposed as low-cost bridge inspection methods. The suggested methods can be divided into two categories. One is direct monitoring, in which sensors are installed directly on the bridges. This method uses multiple sensors on each bridge to measure vibration data. Based on the measured data, the modal parameters of the bridge are estimated, and the bridge’s state is monitored. However, this method requires the installation of multiple sensors on each bridge, which is costly.

**Figure 1:**
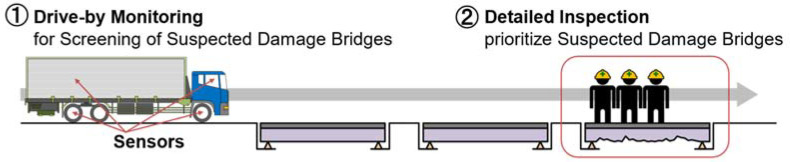
Conceptual diagram of drive-by monitoring.

Indirect monitoring has been proposed as an alternative to costly direct monitoring methods [4]. Indirect monitoring uses mounting sensors on vehicles; the data are measured when vehicles pass over a bridge. The vehicle acts as a bridge exciter and vibration receiver in this method. When a vehicle passes over a bridge, it is shaken by the vehicle. At the same time, the vehicle is also shaken by the bridge. This interaction is called vehicle–bridge interaction (VBI). The vehicle vibration includes the response due to this interaction. Therefore, it is possible to estimate the structural parameters of the bridge from the measured vehicle vibration. Once the structural parameters of the bridge are estimated, priorities for bridge inspection can be determined. Indirect monitoring can be implemented quickly and inexpensively because it is sufficient to mount sensors only on the vehicle. A conceptual diagram of the efficiency of detailed inspections with drive-by monitors is shown in Figure 1.

Existing drive-by-bridge monitoring techniques are affected by road surface irregularities, limited VBI time, and the environment [5]. Therefore, crowdsourcing information collection and the integrated analysis of multiple data runs are considered [6]. Integrated data analysis can eliminate influences unrelated to bridge damage, such as measurements and factors that may affect the behavior of bridges. However, existing methods separately estimate the mass, damping, and stiffness of bridges and road unevenness. Therefore, an analytical method capable of integrated analysis is needed to detect bridge damage from vehicle vibration.

The authors proposed the VBI system identification (VBISI) method [7,8,9]. The VBISI method can simultaneously estimate vehicles’ and bridges’ mechanical parameters and road unevenness from vehicle vibration and location information, under the condition that both the wheelbase and total weight of the vehicle are known. The VBISI method is inspired by technology that simultaneously estimates the vehicles’ mechanical parameters and road unevenness [10,11]. The numerical simulation of bridge vibration is combined with methods proposed in previous studies. The numerical validation of the VBISI method [7,8,9] has already shown the feasibility of this method. However, it is not verified in a field test.

This study aims to identify issues based on the results of field tests of the VBISI method. A Kalman filter is used to mitigate the effects of measurement noise in vehicle vibration.

The contribution of this research is as follows.

- The Kalman filter can be used to estimate road unevenness, but estimating mechanical parameters is challenging.
- The applicability of the VBISI method was verified using experimental data from a 14-t truck and an actual concrete bridge with a 30 m span.
- Through the VBISI method in the field study, a vehicle response analysis problem was proposed.

This paper is organized as follows: Section 2 introduces the related techniques of the VBISI method. Section 3 presents the identification method for the coupled vehicle–bridge system. Section 4 describes the basic mathematics of vehicle–bridge interaction system identification. Next, Section 5 verifies the effectiveness of applying the findings of previous studies to this technology through numerical simulations. Section 6 analyzes the field test data, and Section 7 discusses this study’s limitations and future challenges. Section 8 summarizes the conclusions of this paper.

## 2. Related Work

This section reviews previous studies. Fortunately, there is an extensive review article [5,6,12,13,14,15]. Malekjafarian et al. [5] provided the first comprehensive review of bridge monitoring using vehicle response. Yang et al. [12] summarized methods for estimating the bridge mode shapes and detecting damage from vehicle vibration. Sholravi, H. et al. [13] outlined the broad framework of vehicle-assisted monitoring, which considers the vehicle classification-based SHM from bridge vibration. Hou et al. [14] comprehensively summarized SHM for bridges from 2010 to 2019 and touched on the challenges of damage detection by drive-by monitoring. Based on the previous review paper [5], Malekjafarian et al. [6] discussed drive-by monitoring by dividing it into individual and multiple passes.

### 2.1. Estimating the Bridge Modal Parameter

The estimation of the bridge’s modal parameters is a central topic in indirect monitoring. Modal parameters of bridges are mainly natural frequency, damping, and mode shape. Since indirect monitoring was proposed, identifying bridge frequencies [16,17,18,19,20,21,22,23,24,25,26,27] from vehicle vibrations has been an exciting topic. Yang et al. [4] proposed the first method to estimate the natural frequencies of bridges from vehicle vibrations. After that, Lin and Yang [16] showed applicability in a field test, and Yang and Lin [17] also investigated the estimation of higher-order frequencies. Jian et al. [25] focused on coupled vibration in a 3D model of the vehicle–bridge interaction system. By taking the difference in the acceleration of the front and rear wheels in the frequency domain, it is possible to suppress the influence of road unevenness and identify the bridge’s natural frequency. In addition, estimations of mode shapes [28,29,30,31,32,33,34,35,36] and damping [37,38,39,40,41,42,43,44] were also performed. Yang et al. [35] proposed a method for estimating the bridge mode shapes from the acceleration responses on towed vehicles. Next, the bridge stiffness is calculated from the estimated bridge mode shape. From the estimated bridge stiffness, the bridge deflection was calculated using the finite element method and compared to the measured values [36]. Other methods have been proposed that use machine learning to identify bridge damage from multiple data [45] and estimate bridge vibration from vehicles traveling at high speeds [46]. Yamamoto and Takahashi [47] proposed a damage index that can detect minor damage, such as bolt dropout. Shin et al. [48] proposed a model to discriminate whether the vehicle vibration data were obtained from driving on a bridge or not to implement VBI technology in society.

### 2.2. Estimating the Road Unevenness

Road unevenness also shakes vehicles. If the road pavement is rough, the luggage will deteriorate due to the shaking of the vehicle, and the passenger’s satisfaction will be low. Road roughness affects logistics efficiency, so the World Bank adopted it as an investment decision index. Therefore, it is crucial to manage pavement conditions. Road unevenness reduces the accuracy of estimating bridge modal parameters and damage states from vehicle vibration. Therefore, estimating road unevenness from vehicle vibration is essential. McGetrick et al. [49] proposed a method to estimate road unevenness by calculating dynamic vehicle forces from vehicle vibrations. A study of bridge span lengths and vehicle speeds was conducted to verify the method’s robustness. He and Yang [50] proposed a method for estimating road unevenness on a bridge from a single vehicle using a Kalman filter. The proposed method showed robust results against VBI, vehicle speed, noise contained in vehicle vibration, and bridge damping. Yang et al. [51] estimated the displacement input to the vehicle system (input profile in this study) using the Kalman filter from the measured vehicle vibration. The input profile is the sum of bridge vibration and road unevenness at the axle position. The bridge vibration component is obtained from the difference between the input profile’s front and rear axle positions. Road unevenness can be estimated by subtracting the bridge vibration from the input profile. Hasegawa et al. [52] proposed a road unevenness estimation method using regularized least squares minimization by dynamic programming. Compared to the Kalman filter, it is helpful to use fewer hyperparameters for optimization. The methods described above aim at estimating road unevenness from vehicle vibration.

### 2.3. Estimating the Mechanical Parameters and the Road Unevenness

On the other hand, methods for estimating mechanical vehicle parameters simultaneously with estimating road unevenness have been proposed. Keenahan et al. [53] proposed a method for estimating road unevenness from the vibrations of multiple vehicles traveling on the same route. Their research is interesting because it proposes a method that can integrally process multiple vehicles and driving data. The proposed method was verified by numerical simulations and can also estimate vehicle parameters. Previous research [10,11] estimated vehicles’ mechanical parameters and road unevenness using a Kalman filter from vehicle vibration data measured by smartphones. The smartphone is installed in only one vehicle, and in field tests, road unevenness has been estimated with high accuracy. Techniques for simultaneously identifying road unevenness and vehicles’ mechanical parameters from vehicle vibrations are susceptible to noise. Therefore, it is necessary to reduce the influence of noise using a Kalman filter or the like. The noise reduction method means it is possible to estimate the flatness of a road surface even when the vehicle vibration contains high noise [10,11]. However, when the Kalman filter is used, the accuracy of estimating the mechanical parameters of a vehicle decreases. In particular, the VBISI method can simultaneously estimate a bridge’s mass, damping, and stiffness parameters. However, papers have yet to investigate the relationship between the Kalman filter and the accuracy of estimating the mechanical parameters of vehicles and bridges. In the context of comparison with the VBISI method, the methods for estimating road surface irregularities from vehicle vibrations are summarized in Table 1. The first three columns in Table 1 depict the targets estimated by the methods proposed in each paper. They also distinguish whether the model includes bridges, and whether the estimation process incorporates the Kalman filter. If the relevant condition is satisfied, it is indicated by a symbol ○.

## 3. Preliminaries

Before explaining the VBISI method, this section mentions what a VBI system is.

### 3.1. Vehicle–Bridge Interaction System

VBI systems consist of bridge and vehicle systems. The bridge system takes the contact-point force of a vehicle as an input and returns bridge vibration as an output. Bridge vibration as an output is the response at a fixed point of a bridge. Now, vibrations at fixed points are converted into vibrations at moving points using interpolation. In this paper, the bridge vibration at a moving point is called the bridge profile. On the other hand, the vehicle system receives the input profile and returns the vehicle vibration as an output. The input profile is the sum of the road profile and the bridge profile. Here, the road profile represents road unevenness at the moving point. The vehicle’s contact-point force is obtained from the output vehicle vibration, which is used as the input of the bridge system. In summary, the vehicle and bridge systems have mutual input and output relationships. The VBI system is nonlinear because of repeated interactions as vehicles travel over bridges.

### 3.2. Vehicle–Bridge Interaction System Identification Method

Next, an overview of the VBISI method to be verified in this study is provided. The VBISI method first assumes random vehicle and bridge mechanical parameters. The VBISI method is divided into two processes. The first is the vehicle system’s IEP (input estimation problem) from the vehicle vibration. In this research, the input profile is estimated using the Kalman filter from the equation of motion of the vehicle. The second is the bridge system’s DRS (dynamic response simulation). The input is the contact-point force calculated from the measured vehicle responses. In DRS, the dynamic response of the bridge is calculated by numerical schemes. The bridge profile is obtained from the obtained bridge vibration. The road profile is given by subtracting the bridge profile from the input profile. The obtained road profile can also be converted back to road unevenness. Assuming the vehicle’s pathway is straight, the front and rear wheels run on the same road. However, because the mechanical parameters of the vehicle and bridge are assumed randomly, the estimated road unevenness usually does not match. The objective function is the residual of the road surface roughness estimated for the front and rear wheels. The dynamic parameters of the vehicle and bridge are estimated by solving an optimization problem to minimize this objective function.

## 4. Methodology

The VBISI method verified in this study simultaneously estimates road unevenness and all mechanical parameters (mass, damping, stiffness) both of vehicle and bridge only from the position and vibration data of a traveling vehicle. The formulas of the VBISI method have already been published [7,8,9].

### 4.1. Overview of Vehicle–Bridge Interaction

**Figure 2:**
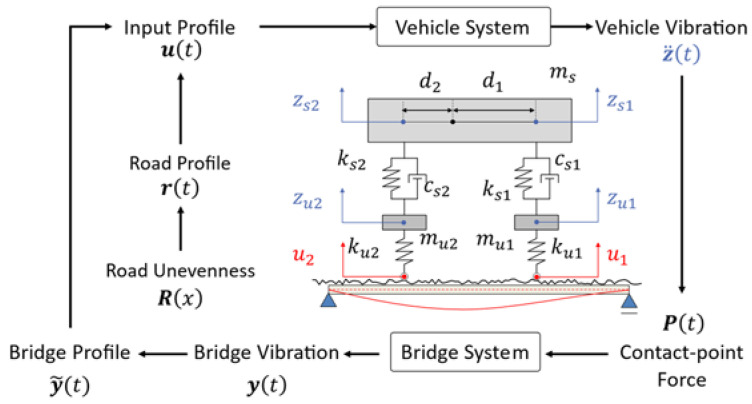
Conceptual diagram of vehicle–bridge interaction [9].

Figure 2 shows a conceptual diagram of the VBI system used in this study. A half-car model is adopted for the vehicle, and the bridge is a simple one-dimensional beam model that considers only bending. The half-car model considers four independent degrees of freedom: the translation and rotation of the car body and the translation of the half-car axle. As the VBI system is a non-linear system and the input/output of the vehicle is the output/input of the bridge, convergence calculation is required to reproduce the vehicle vibration. First, input displacement is given to the vehicle model to obtain the vehicle vibration, and the contact-point force calculated from the vehicle vibration is input to the bridge model to obtain the bridge vibration. The input displacement of the vehicle is updated from the obtained bridge vibration, and the vehicle vibration is calculated again. This process is repeated until the vehicle vibration converges.

### 4.2. Vehicle

For the half-car model shown in Figure 2, $m_{s}$ is the mass of the vehicle body and $c_{si}$, $k_{si}$, $d_{i}$, $m_{ui}$, and $k_{ui}$ represent the suspension damping, the suspension stiffness, the distance from the gravity point, the unspring-mass, and the tire stiffness of the i-th axle, respectively. Let $z_{si}$ be the vertical displacement of sprung-mass vibration at the axle, $z_{si}$ be the unsprung mass, $u_{i}$ be the input profile at the axle. The sprung mass models the vehicle body, and the unsprung mass models the tires and axles. The subscript i corresponds to the axle, with 1 being the front wheel and 2 being the rear wheel. The equation of motion of the vehicle is expressed as [7]

(1) $$M_{v}\ddot{z}\left(t\right)+C_{v}\dot{z}\left(t\right)+K_{v}z\left(t\right)=F_{v}$$

where t  represents the time. $\ddot{z}\left(t\right)$ and $\dot{z}\left(t\right)$ represent the second and first derivatives of $z\left(t\right)$, which are the velocity vibration and the acceleration vibration. $z\left(t\right)$ and $F_{v}\left(t\right)$ can be represented as

(2) $$z\left(t\right)=\left\{\begin{array}{c} z_{s1}\left(t\right)\\ z_{s2}\left(t\right)\\ z_{u1}\left(t\right)\\ z_{u2}\left(t\right) \end{array}\right\}$$

(3) $$F_{v}\left(t\right)=\left\{\begin{array}{c} 0\\ 0\\ k_{u1}u_{1}\left(t\right)\\ k_{u2}u_{2}\left(t\right) \end{array}\right\}$$

(4) Mv=d2msd1+d2d1msd1+d2Id1+d2−Id1+d2  mu1  mu2

(5) $$C_{v}=\left[\begin{array}{cc} \begin{array}{cc} c_{s1} & c_{s2}\\ d_{1}c_{s1} & -d_{2}c_{s2} \end{array} & \begin{array}{cc} -c_{s1} & -c_{s2}\\ -d_{1}c_{s1} & d_{2}c_{s2} \end{array}\\ \begin{array}{cc} -c_{s1} & 0\\ 0 & -c_{s2} \end{array} & \begin{array}{cc} c_{s1} & 0\\ 0 & c_{s2} \end{array} \end{array}\right]$$

(6) $$K_{v}=\left[\begin{array}{cc} \begin{array}{cc} k_{s1} & k_{s2}\\ d_{1}k_{s1} & -d_{2}k_{s2} \end{array} & \begin{array}{cc} -k_{s1} & -k_{s2}\\ -d_{1}k_{s1} & d_{2}k_{s2} \end{array}\\ \begin{array}{cc} -k_{s1} & 0\\ 0 & -k_{s2} \end{array} & \begin{array}{cc} k_{s1}+k_{u1} & 0\\ 0 & k_{s2}+k_{u2} \end{array} \end{array}\right]$$

$M_{v}$, $C_{v}$, and $K_{v}$ are the mass, damping, and stiffness matrices of the vehicle. If the center of rotation coincides with the gravity point, it is known that

(7) $$I=m_{s}d_{1}d_{2}$$

### 4.3. Bridge

Let flexural rigidity and mass per unit length of bridge be $EI\left(x\right)$ and $\rho A\left(x\right)$, respectively; the equation of motion of the bridge system can be expressed as

(8) $$\rho A\ddot{y}\left(x,t\right)+\frac{\partial^{2}}{\partial x^{2}}EI\left(\frac{\partial^{2}}{\partial x^{2}}y\left(x,t\right)\right)=p\left(x,\;t\right)$$

where $y\left(x,t\right)$ denotes the deflection and x represents the position. The external force p consists of the contact-point force $P_{i}\left(t\right)$ of the vehicle and the reaction forces $R_{A}$ and $R_{B}$ at both supports. x=0 indicates the bridge entrance, and x=L indicates the exit. The bridge span length is L. Let the positions of the fulcrums be also $x_{A}=0$ and $x_{B}=L$, respectively, and the position of the i-th wheel be $x_{i}\left(t\right).\;$ The function $\delta \left(x\right)$ represents Dirac’s Delta function. The external force p is expressed as follows [7].

(9) $$p=\overset{2}{\underset{i=1}{\sum }}\delta \left(x-x_{i}\right)P_{i}\left(t\right)+\delta \left(x\right)R_{A}+\delta \left(x-L\right)R_{B}$$

This study applies the finite element method to solve Equation (8) numerically. The finite element formulation is derived by the WRM (weighted residual method). The weighted residual formula in Equation (8) is

(10) $$\int_{0}^{L}\omega \left(\rho A\frac{\partial^{2}y}{\partial t^{2}}+EI\frac{\partial^{4}y}{\partial x^{4}}-p\right)dx=0$$

Let ω be the weight. The weak form of Equation (10) is given by Equation (11).

(11) $$\int_{0}^{L}\left(\rho A\omega \frac{\partial^{2}y}{\partial t^{2}}+EI\frac{\partial^{2}\omega }{\partial x^{2}}\frac{\partial^{2}y}{\partial x^{2}}-p\right)dx=0$$

A one-dimensional finite element model discretizes the bridge vibration $y\left(x,t\right)$ with a Hermite basis.

(12) $$\left\{\begin{array}{c} \phi_{1}\left(s\right)=\frac{1}{4}\left(s-1\right)\left(s-1\right)\left(s+2\right)\\ \phi_{2}\left(s\right)=\frac{\Delta x}{8}\left(s-1\right)\left(s-1\right)\left(s+1\right)\\ \phi_{3}\left(s\right)=-\frac{1}{4}\left(s+1\right)\left(s+1\right)\left(s-2\right)\\ \phi_{4}\left(s\right)=\frac{\Delta x}{8}\left(s-1\right)\left(s+1\right)\left(s+1\right) \end{array}\right.$$

where s represents the normalized local coordinate in each element. Assuming that the j-th and $\left(j+1\right)$-th nodes compose the j-th beam elements, X=−1 indicates the position of the j-th node, and X=1 indicates the position of the $\left(j+1\right)$-th node. $\Delta x=x_{j}-x_{j+1}$ when the whole system is inside beam element j, which consists of node $x_{j}$ and node $x_{j+1}$. Define a basis function vector $N\left(x\right)$ whose components are

(13) $$\left\{\begin{array}{c} N_{2j-1}\left(x\right)=\phi_{1}\left(s\right)\\ N_{2j+0}\left(x\right)=\phi_{2}\left(s\right)\\ N_{2j+1}\left(x\right)=\phi_{3}\left(s\right)\\ N_{2j+2}\left(x\right)=\phi_{4}\left(s\right) \end{array}\right.$$

All components outside the element are set to zero. Using the bridge deflection $y\left(x_{j},t\right)\;$ and deflection angle $\theta \left(x_{j},t\right)\;$ at the nodes, the deformation vector $y\left(t\right)\;$ is

(14) $$\left\{\begin{array}{c} y_{2\left(j-1\right)+1}\left(t\right)=y\left(x_{j},t\right)\\ y_{2\left(j-1\right)+2}\left(t\right)=\theta \left(x_{j},t\right) \end{array}\right.$$

Then, the approximate solution of $y\left(x,t\right)$ is

(15) $$y\left(x,t\right)=N\left(x\right)\cdot y\left(t\right)$$

Similarly, by setting the weights to $\omega \left(x\right)=N\left(x\right)\cdot \omega$ and substituting them into Equation (11), we obtain the following.

(16) $$\omega^{T}\left(M_{b}\ddot{y}\left(t\right)+K_{b}y\left(t\right)-F\left(t\right)\right)=0$$

$M_{b}$, $C_{b}$, and $K_{b}$ are the mass, damping, and stiffness matrices of the bridge.

(17) $$M_{b}=\int_{0}^{L}NN^{T}dx$$

(18) $$K_{b}=\int_{0}^{L}\frac{\partial^{2}N}{\partial x^{2}}\frac{\partial^{2}N^{T}}{\partial x^{2}}dx$$

$F\left(t\right)$ is an external force vector whose components are the external forces (concentrated load and moment of force) at each node. Considering Rayleigh damping,

(19) $$C_{b}=\alpha M_{b}+\beta K_{b}$$

Solving for the integration condition in Equation (16) for any ω gives the following finite element equation.

(20) $$M_{b}\ddot{y}\left(t\right)+C_{b}\dot{y}\left(t\right)+K_{b}y\left(t\right)=F\left(t\right)$$

### 4.4. Vehicle–Bridge Interaction

In general, the responses of vehicles and bridges are modeled by interactions with each other’s outputs as inputs. In order to realize a numerical simulation considering this interaction, the following steps are performed. First, the vehicle vibration is calculated by inputting only the road profile. Then, the contact-point force to the bridge is obtained from the obtained vehicle vibration, and the bridge vibration is calculated. Adding this bridge profile to the road profile creates a new input profile, and the vehicle vibration is obtained again. By repeating this process, the displacement vibration of the vehicle and the bridge is obtained. The input profile and contact-point force, which are the inputs of the vehicle and bridge, are explained below [7].

#### 4.4.1. Input Profile

The input profile $u\left(t\right)$, which is the input of the vehicle system, is given by the sum of the road profile $r\left(t\right)$ and the bridge profile $\tilde{y}\left(t\right)\;$ and is expressed by Equation (21).

(21) $$u\left(t\right)=r\left(t\right)+\tilde{y}\left(t\right)$$

Here, the road profile represents the road at the axle position. When the road unevenness is $R\left(x\right)$ and the axle position is $x_{i}\left(t\right)$,

(22) $$r_{i}\left(t\right)=R\left(x_{i}\left(t\right)\right)$$

$r_{i}\left(t\right)$ is the component of $r\left(t\right)$. On the other hand, the bridge profile is the bridge vibration $y\left(x,t\right)\;$ at the axle position $x_{i}\left(t\right)$. In other words, the bridge vibration is the deformation vector of each node fixed on the bridge. Therefore, converting to bridge displacement at the axle position is necessary. The same basis used in the discretization was used. The transformation matrix is

(23) $$L\left(t\right)=\left[\begin{array}{cc} N\left(x_{1}\left(t\right)\right) & N\left(x_{2}\left(t\right)\right) \end{array}\right]$$

The bridge profile is

(24) $$\tilde{y}\left(t\right)=L^{T}\left(t\right)y\left(t\right)$$

#### 4.4.2. Contact-Point Force

The contact-point force, which is the input to the bridge, corresponds to the restoring force acting on the tire. However, as the equation of motion of the vehicle (Equation (1)) is based on the equilibrium position, it should be noted that the gravity term has disappeared. In calculating the restoring force, the effect of gravity is considered based on the equilibrium length. Considering that the center of gravity of the vehicle body is the center of gravity, the contact-point force between the front and rear wheels is

(25) $$V_{1}\left(t\right)=-\frac{d_{2}m_{s}}{d_{1}+d_{2}}\left(g+{\ddot{z}}_{s1}\right)-m_{u1}\left(g+{\ddot{z}}_{u1}\right)\;V_{2}\left(t\right)=-\frac{d_{1}m_{s}}{d_{1}+d_{2}}\left(g+{\ddot{z}}_{s2}\right)-m_{u2}\left(g+{\ddot{z}}_{u2}\right)$$

The external force vector acting on the bridge is

(26) $$F\left(t\right)=L\left(t\right)\left[\begin{array}{cc} V_{1}\left(t\right) & V_{2}\left(t\right) \end{array}\right]+H\left(t\right)$$

$H\left(t\right)$ represents the fulcrum reaction force.

### 4.5. System Identification

It is assumed that vehicle vibration is obtained as measurement data. Here, the road profile can be estimated by substituting the vehicle vibration data and the vehicle and bridge parameters whose initial values are randomly assumed in the VBI system. By positionally synchronizing the obtained road profile, the estimated road unevenness $R_{1}\left(x\right)$ and $R_{2}\left(x\right)$ for the front and rear wheels can be obtained. When the vehicle travels straight, the front and rear wheels are considered to run on the same road unevenness. Therefore, the estimated road unevenness $R_{1}\left(x\right)$ and $R_{2}\left(x\right)$ should also match. However, as the mechanical parameters are given randomly, they do not match. Therefore, the optimization problem is to update the dynamic parameters to minimize the error between the estimated road unevenness $R_{1}\left(x\right)$ and $R_{2}\left(x\right)$. In other words, the VBISI method is a search method for mechanical parameters where $R_{1}\left(x\right)$ = $R_{2}\left(x\right)$.

#### 4.5.1. Kalman Filter

In order to estimate the road unevenness from the vehicle vibration, this study also introduced the discrete-time extended state-space model proposed by Xu et al. [10]. The following equations give the state vector and observation vector.

(27) $$Z_{k}=\left\{\begin{array}{c} z\left(k\Delta t\right)\\ \dot{z}\left(k\Delta t\right)\\ u\left(k\Delta t\right)\\ \dot{u}\left(k\Delta t\right) \end{array}\right\}$$

(28) $$s_{k}=\left\{\begin{array}{c} \ddot{z}\left(k\Delta t\right)\\ z\left(k\Delta t\right) \end{array}\right\}$$

The state vector is given by the vehicle’s vertical displacement vibration, velocity vibration, input profile, and velocity vibration. The discrete-time extended state-space model in this study can be written as follows:

(29) $$Z_{k}=\bar{V}Z_{k-1}+\omega_{k}$$

(30) $$s_{k}=HZ_{k}+\epsilon_{k}$$

The matrix index $\bar{V}$ is given by Equation (31), where $\omega_{k},\epsilon_{k}$ represent system noise and observation noise, and $\omega \sim N\left(0,Q\right),\epsilon \sim N\left(0,R\right)$.

(31) $$\bar{V}=\mathrm{expm}\left[V\Delta t\right]\;=U\mathrm{diag}\left(\exp\left(D\right)\right)U^{-1}$$

(32) $$V=\left[\begin{array}{cccc} O^{4\times 4} & I^{4\times 4} & O^{4\times 2} & O^{2\times 2}\\ -M_{v}^{-1}K_{v} & -M_{v}^{-1}C_{v} & M_{v}^{-1}F_{v} & O^{2\times 2}\\ O^{2\times 4} & O^{2\times 4} & I^{2\times 2} & O^{2\times 2}\\ O^{2\times 4} & O^{2\times 4} & O^{2\times 2} & O^{2\times 2} \end{array}\right]$$

U and D are the modal matrices and diagonal matrix when VΔt is diagonalized and are expressed as follows.

(33) $$V\Delta t=UDU^{-1}$$

Additionally, **H** is given by

(34) $$\;H=[\begin{array}{cccccccccccc} -\frac{k_{s1}}{m_{s}} & 0 & \frac{k_{s1}}{m_{s}} & 0 & -\frac{c_{s1}}{m_{s}} & 0 & \frac{c_{s1}}{m_{s}} & 0 & 0 & 0 & 0 & 0\\ 0 & -\frac{k_{s2}}{m_{s}} & 0 & \frac{k_{s2}}{m_{s}} & 0 & -\frac{c_{s2}}{m_{s}} & 0 & \frac{c_{s1}}{m_{s}} & 0 & 0 & 0 & 0\\ \frac{k_{s1}}{m_{u1}} & 0 & -\frac{\left(k_{s1}+k_{u1}\right)}{m_{u1}} & 0 & \frac{c_{s1}}{m_{u1}} & 0 & -\frac{c_{s1}}{m_{u1}} & 0 & \frac{k_{u1}}{m_{u1}} & 0 & 0 & 0\\ 0 & \frac{k_{s1}}{m_{u2}} & 0 & -\frac{\left(k_{s2}+k_{u2}\right)}{m_{u2}} & 0 & \frac{c_{s2}}{m_{u2}} & 0 & -\frac{c_{s1}}{m_{u2}} & 0 & \frac{k_{u2}}{m_{u2}} & 0 & 0\\ 1 & 0 & 0 & 0 & 0 & 0 & 0 & 0 & 0 & 0 & 0 & 0\\ 0 & 1 & 0 & 0 & 0 & 0 & 0 & 0 & 0 & 0 & 0 & 0\\ 0 & 0 & 1 & 0 & 0 & 0 & 0 & 0 & 0 & 0 & 0 & 0\\ 0 & 0 & 0 & 1 & 0 & 0 & 0 & 0 & 0 & 0 & 0 & 0 \end{array}]$$

Let Q and R be the variance–covariance matrices of system noise $\omega_{k}$ and observation noise $\epsilon_{k}$.

(35) $$Q=E\left[\omega_{k}\omega_{k}^{T}\right]$$

(36) $$R=E\left[\epsilon_{k}\epsilon_{k}^{T}\right]$$

Apply the Kalman filter [54] to the obtained discrete-time extended state-space model. According to Xue et al. [10], it is possible to estimate the input profile even in a noisy environment. In addition, the Robbins–Monro algorithm [55] can be used to dynamically estimate the variance–covariance matrix of process noise and observation noise. Xue et al. [10] also used Rauch-Tung-Striebel smoothing [56], which was also applied in this study. The Kalman filter [55] is an efficient method for obtaining state vectors based on observed data and a dynamic model. Let the estimated state vector be ${\hat{Z}}_{k}$. Now, when ${\hat{Z}}_{k-1}$ is obtained, the candidate X of ${\hat{Z}}_{k}$ from Equation (29) is $X\sim N\left(\mu_{a},\Sigma_{a}\right)$. On the other hand, when $s_{k}$ is obtained, the candidate Y of ${\hat{Z}}_{k}$ derived from Equation (30) follows $Y\sim N\left(\mu_{b},\Sigma_{b}\right)$.

(37) $$\mu_{a}=\bar{V}{\hat{Z}}_{k-1}$$

(38) $$\Sigma_{a,k}=\bar{V}P_{k-1}{\bar{V}}^{T}+Q$$

(39) $$\mu_{b}=H^{-1}s_{k}$$

(40) $$\Sigma_{b}=H^{-1}RH^{-T}$$

After applying the Kalman filter, the state vector ${\hat{Z}}_{k}$ is estimated as the maximum likelihood value, assuming that the two candidates X and Y follow a normal distribution. That is, ${\hat{Z}}_{k}$ is derived as follows.

(41) $$\begin{array}{l} {\hat{Z}}_{k}={\left(\Sigma_{a,k}^{-1}+\Sigma_{b}^{-1}\right)}^{-1}\left(\Sigma_{a,k}^{-1}\mu_{a}+\Sigma_{b}^{-1}\mu_{b}\right)\\ \;\;\;\;\;=\left[I-G_{k}H\right]\left\{\bar{V}{\hat{Z}}_{k-1}\right\}+\left[G_{k}H\right]\left\{H^{-1}s_{k}\right\}\\ \;\;\;\;\;=\left[I-G_{k}H\right]\left\{\bar{V}{\hat{Z}}_{k-1}\right\}+G_{k}s_{k} \end{array}$$

where $G_{k}$ and $P_{k}$ are calculated as follows.

(42) $$G_{k}=\Sigma_{a,k}H^{T}{\left(H\Sigma_{a,k}H^{T}+R\right)}^{-1}$$

(43) $$P_{k}={\left(\Sigma_{a,k}^{-1}+\Sigma_{b}^{-1}\right)}^{-1}=\left[I-GH\right]\Sigma_{a}$$

Subsequently, the Robbins–Monro algorithm [55] dynamically estimates the variance–covariance matrix of the process noise $\omega_{k}$ and the observation noise $\epsilon_{k}$. Correctly estimating the order of noise is important because it affects the practicality of Kalman filter-based road profile estimation. Here, $\alpha_{Q,k}$ and $\alpha_{R,k}$ are positive real numbers smaller than one.

(44) $$Q_{k}=\left(1-\alpha_{Q,k}\right)Q_{k-1}+\alpha_{Q,k}G_{k}\left(s_{k}-H{\hat{Z}}_{k}\right){\left(s_{k}-H{\hat{Z}}_{k}\right)}^{T}G_{k}^{T}$$

(45) $$R_{k}=\left(1-\alpha_{R,k}\right)R_{k-1}+\alpha_{R,k}\left(s_{k}-H{\hat{Z}}_{k}\right){\left(s_{k}-H{\hat{Z}}_{k}\right)}^{T}$$

#### 4.5.2. Object Function

First, $\dot{z}\left(t\right)$ and $z\left(t\right)$ are obtained by applying the Newmark-*β* method to vehicle vibration $\ddot{z}\left(t\right)$, which is the measurement data. $M_{v}$, $C_{v}$, and $K_{v}$ can also be obtained by randomly assuming the system parameters of the vehicle. At this time, $u_{1}$ and $u_{2}$ can be obtained by estimating the state vector using the Kalman filter. Next, the vehicle vibration data $\ddot{z}\left(t\right)$ and the assumed vehicle system parameters are substituted into Equation (25) to obtain contact-point forces $V_{1}\left(t\right)$ and $V_{2}\left(t\right)$. Suppose the system parameters of the bridge are also assumed randomly. In that case, the bridge vibration $y\left(t\right)$ can be obtained using the equation of motion of the bridge and the Newmark-*β* method, as in numerical simulation. By substituting into Equation (24), the bridge profile $\tilde{y}\left(t\right)$ can be obtained. Then, **r**(t) can be estimated by subtracting $\tilde{y}\left(t\right)$ from u(t) obtained earlier. Here, $R_{1}\left(x\right)$ is obtained by synchronizing $r_{1}\left(t\right)$ with $x_{1}\left(t\right)$, and $R_{2}\left(x\right)$ is obtained by synchronizing $r_{2}\left(t\right)$ with $x_{2}\left(t\right)$. Based on the assumption that road unevenness $R_{1}\left(x\right)$ and $R_{2}\left(x\right)$ should be equal, the problem of estimating mechanical parameters is treated as an optimization problem that minimizes the squared error of $R_{1}\left(x\right)$ and $R_{2}\left(x\right)$. The objective function of this optimization problem is

(46) $$J\left(x\right)=\sum {\left|R_{1}\left(x\right)-R_{2}\left(x\right)\right|}^{2}$$

If the parameters of the vehicle and bridge are all correct values, the two road unevenness calculations match. Therefore, if the parameters can be updated so that the calculated road unevenness matches, it can be expected that the parameters will eventually approach the correct values. However, the equations of motion are equivalent when all parameters are multiplied by the same factor. Therefore, at least one parameter must be known. This research assumes that the gross vehicle weight $M=m_{s}+m_{u1}+m_{u2}$ and the distance between axles $D=d_{1}+d_{2}$, which are easy to measure, are known parameters [7].

#### 4.5.3. Optimization Method

This research adopts the Nelder–Mead method [57,58] as the parameter search method. The method creates an initial simplex (a simulated triangle in high-dimensional space). The simplex is then iteratively modified to approach the minimum or maximum value of the function. The simplex is then modified using the reflection, expansion, contraction, and shrink methods. The Nelder–Mead method is independent of the slope of the objective function and searches for the optimal solution relatively quickly. This study uses the faster adaptive Nelder-Mead method [58]. In addition, there are non-negative conditions and constraints on mechanical parameters. Therefore, the objective function of the VBISI method is changed from a constrained objective function to an unconstrained objective function using a penalty function. The penalty function sets the value of the objective function to infinity if the parameters do not satisfy the following conditions.

1. One of the parameters is negative.
2. The sum of axle weights $m_{u1}$ and $m_{u2}$ exceeds the vehicle weight.
3. The center of gravity position $d_{1}$ exceeds the wheelbase value.

For the initial value, give 0.8 to 1.2 times the value assumed in advance.

### 4.6. Implementation of Numerical Simulation

Based on the model shown in Figure 2, the coupled vibration of the vehicle and bridge is reproduced by numerical simulation. Here, the vehicle model is separated from the bridge model, and the computation is repeated until the vehicle vibration converges [7].

#### 4.6.1. Newmark-β Method

Vehicle and bridge vibrations are obtained by applying the Newmark-β method to the respective equations of motion. The arbitrary equations of motion are shown below.

(47) $$M\ddot{\eta }\left(t\right)+C\dot{\eta }\left(t\right)+K\eta \left(t\right)=\xi \left(t\right)$$

Discretize the time function $\eta \left(t\right)$, and let $\eta_{k}$ be the displacement response of the vehicle or bridge, where Δt is the time increment

(48) $$\eta_{k}=\eta \left(k\Delta t\right).$$

In the Newmark-β method,

(49) $$\begin{array}{l} {\dot{\eta }}_{k}={\dot{\eta }}_{k-1}+\Delta t\left(\left(1-\gamma \right){\ddot{\eta }}_{k-1}+\gamma {\ddot{\eta }}_{k-1}\right)\\ \eta_{k}=\eta_{k-1}+\Delta t{\dot{\eta }}_{k-1}+\Delta t^{2}\left(\left(\frac{1}{2}-\beta \right){\ddot{\eta }}_{k-1}+\beta {\ddot{\eta }}_{k}\right) \end{array}$$

From the above equation, the equation can be written as follows

(50) $$M{\ddot{\eta }}_{k}+C{\dot{\eta }}_{k}+K\eta_{k}=\xi_{k}$$

Applying the Newmark-β method, we receive

(51) $$A{\ddot{\eta }}_{k}=b_{k}.$$

From this, the following can be derived

(52) $$A=\left[M+\Delta t\gamma C+\Delta t^{2}\beta K\right]$$

(53) $$b_{k}=\left\{\xi_{k}-C\left({\dot{\eta }}_{k-1}+\Delta t\left(1-\gamma \right){\ddot{\eta }}_{k-1}\right)-K\left(\eta_{k-1}+\Delta t{\dot{\eta }}_{k-1}+\Delta t^{2}\left(\frac{1}{2}-\beta \right){\ddot{\eta }}_{k-1}\right)\right\}$$

#### 4.6.2. Iterative Computation

Iterative calculations reproduce the coupled vibration of the vehicle and bridge. First, vehicle vibration is calculated using only the road unevenness as an input. The data matrix $Z=\left[\cdots z_{k}\cdots \right]$ represents the discretized vehicle vibration. The obtained vehicle vibration Z is set to $Z^{0}$, as convergence calculations are performed, and using $Z^{0}$, the ground forces can be obtained. Replacing Equation (47) with the equation of motion of the bridge (Equation (20), the bridge vibration is obtained in the same way. Let this be $Y^{0}$ from $Y^{0}$; $Z^{1}$ is obtained. This process is repeated to obtain $Y^{l}$ from $Z^{l}$ and $Z^{l+1}$ from $Y^{l}$. The convergence condition for this iterative calculation is

(54) $$\varepsilon =\frac{\left|Z^{l+1}-Z^{l}\right|}{\left|Z^{l+1}\right|}\leq \varepsilon_{max}.$$

The computation is terminated when the update ratio of $Z^{l}$ is less than the threshold value $\varepsilon_{max}$. Additionally,   denotes the quadratic norm.

The time increment is set to $1.0\times 10^{-3}\left[s\right]$, and the threshold for convergence judgment is set to $1.0\times 10^{-6}$. The Newmark-β method employs the average acceleration method, with $\gamma =\frac{1}{2}$ and $\beta =\frac{1}{4}$.

## 5. Numerical Validation

### 5.1. Setting

First, this study verifies whether road unevenness can be estimated from vehicle vibration calculated by numerical simulation. To see how the Kalman filter’s application changes the parameter estimation accuracy. The mechanical parameters of the vehicle were determined from the vehicle inspection certificate. The vehicle is assumed to be a 14-t truck that can sufficiently vibrate the bridge. The mechanical parameters of the bridge were determined concerning the bridge ledger (Table 2). The roughness of the road profile used in the numerical simulation was determined based on the values calculated by the road profiler. The vehicle displacement vibration is estimated from the acceleration using the *Newmark-*β method. The estimated vehicle displacement vibration is high-pass filtered at 0.1 Hz. The filtering process is to remove trends due to numerical integration.

The authors assumed values for the variance–covariance matrix based on previously measured vehicle vibration data. Table 3 summarizes the initial value diagonal components of the variance–covariance matrix of the process and observation noise. Even if the model parameters corresponding to an actual vehicle are estimated, there will consistently be modeling errors because the half-car model is a simplified linear system with limited degrees of freedom. It is generally difficult to estimate the degree of the first through eighth components of Q corresponding to this modeling error [10]. Therefore, the values were determined by trial and error with reference to [10]. On the other hand, the observed noise value can be determined by referring to the noise level of the measurement equipment and the installation method. However, it is difficult to accurately estimate these values for each measurement vehicle and installation method. Therefore, R was also determined by trial and error. In this study, the values were set based on the RMS values of vehicle vibration measured when the vehicle was stationary; the off-diagonal elements of Q and R are zero.

### 5.2. Result and Discussion

**Figure 3:**
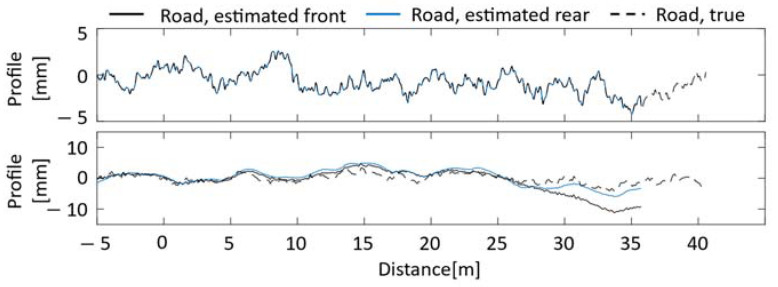
Road unevenness estimation results without Kalman filter [upper: without noise, lower: with noise].

**Figure 4:**
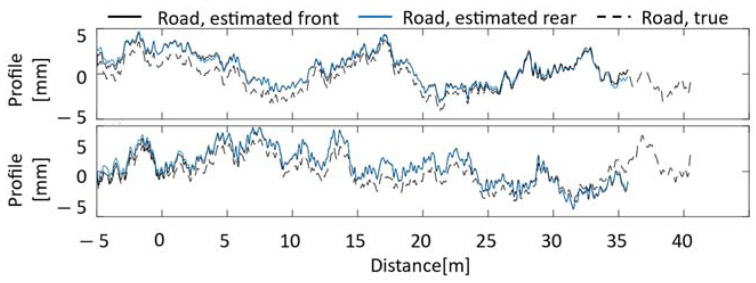
Road unevenness estimation results with Kalman filter [upper: without noise, lower: with noise].

Parameter identification and road unevenness estimation are performed using vehicle vibrations. The effect of the Kalman filter on parameter identification was confirmed through comparison with the case where the Kalman filter was not used. The diagonal elements of the variance–covariance matrices R and Q of the Kalman filter without consideration of noise are assumed to be $1.00\times 10^{-9}$ for all one to eight elements. In addition, the case where noise is included in the vehicle vibration is also considered for field tests. The noise was set based on the value measured by an acceleration sensor installed in the vehicle in a static state. Figure 3 shows the estimation of road unevenness by the VBISI method without a Kalman filter. Figure 4 shows the results using the Kalman filter. The upper and lower rows show the estimated road unevenness when noise is not considered and when noise is considered, respectively. The set vehicle speed is 8.1 [m/s], and the data for about 5 s is used. The Nelder–Mead method was adopted to minimize the objective function, and the number of updates was set to 1000 times. The input profile without the Kalman filter is obtained from Equations (1) and (3).

When the Kalman filter is not used, the road unevenness estimated for the front and rear wheels almost always coincide with the absence of noise. In addition, the road unevenness calculated for the front and rear wheels almost always overlaps with the correct value. However, when noise is considered, the estimation accuracy is significantly reduced. On the other hand, when the Kalman filter is used, the decrease in the accuracy of road unevenness is slight regardless of the presence or absence of noise. However, the road unevenness estimated for the front and rear wheels differs from the correct ones. There are several possible reasons for this. One is that the parameters of the variance–covariance matrix of the Kalman filter need to be better adjusted, which may have been affected by the noise processing. It is also known that the accuracy of the Kalman filter in estimating road unevenness decreases as the distance from the center of gravity increases [7]. There is a possibility that the influence of the estimation error of the front and rear wheels cannot be ignored. Table 4 summarizes the parameter estimation results. The two columns on the left in Table 4 indicate the presence or absence of the Kalman filter in the estimation process and the consideration or lack thereof of noise to vehicle vibration. If the conditions are met, a symbol of ○ is given; if not, a symbol of ✕ is assigned.

Estimated parameters were divided by their correct values and normalized. Only the bridge endpoints and the central part are represented as bridge stiffness values. The highest accuracy is obtained when the Kalman filter is not used and noise is not considered. Even if the Kalman filter is used, it is possible to estimate the mechanical parameters of the vehicle and bridge without considering noise. However, the accuracy of the estimated parameters is slightly lower than before using the Kalman filter. This phenomenon occurs even though the front and rear wheels estimate almost the same road surface. In other words, the difference between the estimated road unevenness and the correct road unevenness is the cause. In addition, when noise is considered, the parameter estimation accuracy decreases in both methods regardless of the introduction of the Kalman filter.

## 6. Field Test

### 6.1. Bridge Description

**Figure 5:**
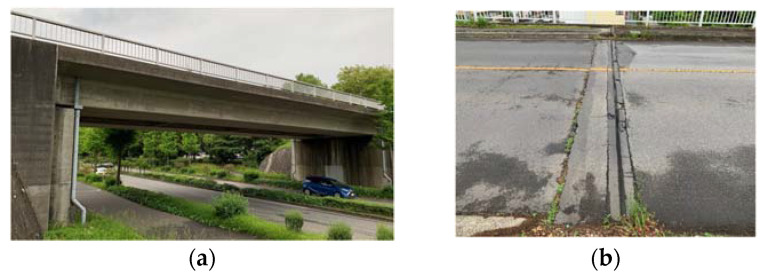
The view of the bridge: (**a**) full view; (**b**) expansion joint [48].

Matsumi Bridge is a one-span bridge constructed in 1973 across Kaede Street in Tsukuba City, Ibaraki Prefecture, Japan (Figure 5). The bridge’s total length is about 30.88 m, and the width is about 12.98 m. The main girder is a PC box post-tension girder. It is paved with asphalt and integrated with the main girder.

To provide a baseline for comparison with drive-by measurements, the natural frequency of Matsumi Bridge is directly measured by installing accelerometers on the bridge. A three-axis wireless MEMS accelerometer was used to record the forced vibration of the bridge. Two sensors were installed at the center of each bridge for bridge measurement. A wireless accelerometer driven by a mobile battery was attached using double-sided tape for construction. Vertical acceleration was measured at 300 Hz.

**Figure 6:**
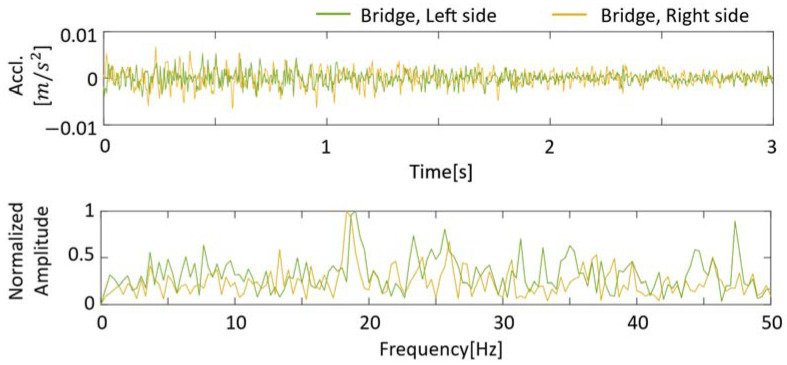
Free-vibration response of the Matsumi Bridge in both the time and frequency domain.

The first natural frequency of Matsumi Bridge was calculated by the fast Fourier transform of free vibration and forced vibration data. Figure 6 shows an example of the free vibration and FFT of the bridge after passing vehicles measured at two locations. Additionally, forced vibration is shown in Figure 6. In this example, a passing measurement vehicle excites a bridge. Comparing the free vibration and vehicle vibration, the latter has more frequencies in the lower frequency range than the fundamental frequency of the bridge (including vehicle frequencies). As shown in Figure 6, the first peak in the frequency domain, corresponding to the fundamental frequency of the bridge, was evident in the data from all two sensors. The averages of five measurements of free vibration and forced vibration due to the passage of the measurement vehicle were 18.6 Hz and 18.33 Hz. The lower frequency of forced oscillation than that of free oscillation can be attributed to the mass increase by the measurement vehicle [59]. As there are only two sensors in this study, it is impossible to determine which bridge mode is responsible for the observed peaks.

### 6.2. Measured Data

**Figure 7:**
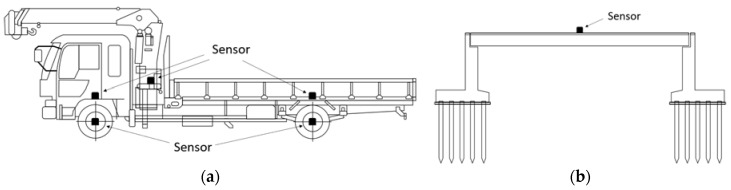
Layout of sensor installation: (**a**) vehicle; (**b**) bridge.

**Figure 8:**
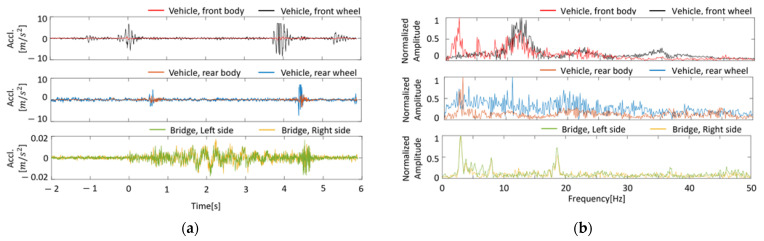
Vibration response of vehicle and bridge: (**a**) in time domain; (**b**) in frequency domain.

Measurements were taken with multiple accelerometers and multiple GPSs for a two-axis vehicle. The layout of the installed sensors is shown in Figure 7. The instrumentation and testing details are presented in [48]. Over 18 and 19 May 2022, the vertical acceleration was measured when the measurement vehicle crossed Matsumi Bridge 100 times. In VBI studies, the measured acceleration vibration is often in the vertical direction. However, it is difficult to obtain data only in the vertical direction when accelerometers are installed in vehicles, depending on the location. Therefore, the acceleration data were corrected using a rotation matrix before conducting this analysis. The accelerometers used in this study were triaxial, and the vertical direction can be inferred in the post-process by correcting the data with a rotation matrix. The sensor tilt was estimated from the vehicle vibration measured while the vehicle was stopped and corrected. The obtained rotation matrix is applied to the vehicle vibration data for analysis to obtain the mean value of the acceleration signal in the vertical direction, and the process of average zeroing is performed. Figure 8 shows an example of the vertical acceleration of a vehicle and the forced vibration of a bridge and its FFT results. The upper, middle, and lower rows represent the vehicle’s front, rear, and bridge vibrations. For vehicle vibration, the vibration of the vehicle body and the vibration of the axle are plotted simultaneously. In common with front and rear vehicle vibrations, the amplitude of axle vibration is larger than vehicle body vibration. In the frequency domain, both the vehicle and bridge vibrations have a peak of about 3 Hz. By VBI, a peak was observed that did not exist during forced vibration (Figure 8b). Some peaks are seen around 13 Hz at the front of the vehicle. These are due to the effects of the vehicle’s natural frequency, engine vibration, and road unevenness. In addition, the FFT of vehicle vibration does not show a peak near 18 Hz. Therefore, it is difficult to identify the fundamental frequency of a bridge simply by the FFT of the measurement data. The Nelder–Mead method was adopted to minimize the objective function, and the number of updates was set to 10,000 times.

### 6.3. Results

**Figure 9:**
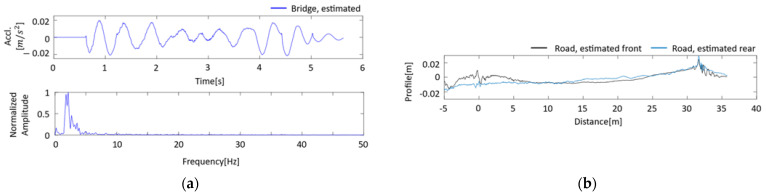
The VBISI method estimation result: (**a**) bridge vibration in time and domain; (**b**) road profile.

**Figure 10:**
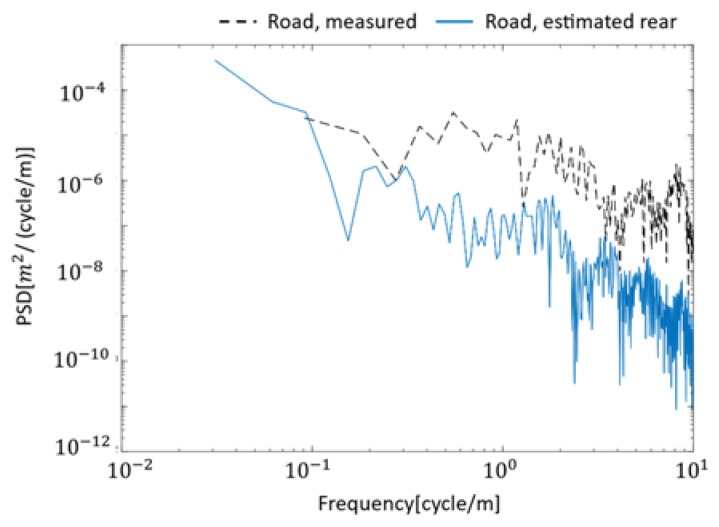
Comparison of estimated and measured PSD functions of road unevenness.

The results of the VBISI method run using the data measured in the field test are summarized in Figure 9 and Figure 10 and Table 4. Figure 9a shows the acceleration vibration of the bridge vibration estimated by the VBISI method, its FFT result, and the estimated road profile. In the frequency domain of Figure 6, a peak around 18 Hz can be observed for the bridge vibration. This peak could not be observed from the estimated bridge vibration, as shown in Figure 9a. Figure 9b shows the road profile estimated by the VBISI method. Large amplitudes can be seen around 0 m and 30 m of the estimated road unevenness. These amplitudes can be attributed to the expansion joint in front of and behind the bridge. The road unevenness estimated for the front and rear wheels show similar characteristics, but they did not match. Figure 10 compares the power spectral density function (PSD) of the VBISI-estimated and measured road unevenness. The estimated road unevenness was assessed at the vehicle’s front axle position. The road unevenness was measured using a road profiler. The estimated road unevenness is lower than the measured road unevenness. Table 5 summarizes the vehicle and mechanical bridge parameters estimated by the VBISI method. The correct values of the modal parameters for vehicles and bridges in a field test have yet to be discovered. Therefore, evaluating the parameters estimated by this method is a technical issue.

## 7. Discussion

When the estimated road unevenness matches the correct values [8,9], the mechanical parameters of the vehicle and bridge also mostly match the correct values. Therefore, the accurate estimation of road unevenness is essential. To improve the estimation accuracy, actions are taken to improve the accuracy of the road unevenness estimation. In this study, several possible factors may reduce the accuracy of road unevenness estimation.

One is the problem of measured data. In this study, vehicle acceleration vibration and displacement vibration are used as observation data. However, vehicle displacement vibration is calculated by the numerical integration of vehicle acceleration vibration. The acceleration vibration of a vehicle contains various noises. Vibrations included in vehicle vibration may include engine vibration. Therefore, the numerically integrated displacement vibration of the vehicle has a trend. Because it is difficult to remove this effect, the accuracy of the road unevenness estimation is reduced. It is possible to use only acceleration vibration as the observed data. However, displacement vibration is essential to satisfy observability in the state-space model. In a model that does not satisfy observability, it is difficult to determine the state variables from the observed data uniquely. On the other hand, the vehicle is also equipped with a GPS sensor to measure vertical displacement. The model’s accuracy can be improved if the GPS’s vertical displacement can compensate for the accelerometer’s displacement oscillations.

Optimization also poses challenges. Many nonlinear optimization problems have multiple local solutions. As the objective function of the VBISI method is also a nonlinear function, there are local solutions. Therefore, even if the optimal solution is obtained, there may be a different value. Moreover, in the absence of blueprints, it is necessary to increase the range of parameters to be explored. Therefore, a more efficient parameter search method is needed for future verification. There is also potential for improvement in the objective function. Although this study used road unevenness estimated from the front and rear wheel positions, the synchronization of the positions takes work.

It is also necessary to consider how to verify the accuracy of this method. Therefore, it is practical to conduct a laboratory experiment to examine the applicability of this method. In laboratory experiments, it is relatively easy to grasp the mechanical parameters of the vehicle and bridge. In addition, it may be possible to estimate mechanical vehicle parameters from free vibration tests using humps and compare them with the results. Mechanical bridge parameters are also being considered to use findings from direct monitoring of bridges. A sensor is installed directly on the bridge, and the excitation test estimates the modal parameters of the bridge. Nikkhoo et al. [60] proposed a method for estimating the natural frequencies and dynamic response of various beams subjected to excitation by a moving mass. If these direct bridge monitoring techniques can be utilized, more efficient and reliable parameter estimation may be possible. Additionally, Yang et al. [36] proposed a method to evaluate the stiffness of a bridge estimated from vehicle vibration. Based on the estimated bridge stiffness, the deflection of the bridge, when given a specific load, is calculated by the finite element method. A similar load is applied to an actual bridge and compared with its displacement to verify accuracy. In addition, the VBISI method does not examine vehicle speed restrictions, which have been pointed out in previous studies [61]. Therefore, it is necessary to verify the practicality of this technique through parametric studies.

## 8. Conclusions

A field study of an indirect bridge health monitoring method for single-span concrete bridges is described. Acceleration responses extracted from sensor-mounted sensors were used. The study examined methods for estimating (i) mechanical vehicle parameters, (ii) mechanical bridge parameters, and (iii) road unevenness. Direct measurements using sensors installed on the bridges confirmed that the vehicles shook the bridge. Road profilers were also used to measure road unevenness. It is difficult to identify the fundamental frequencies of bridges from the estimated bridge vibrations. In addition, the estimated road unevenness needs to be more accurate when compared to the measured road unevenness, although they show some of the same trends. This paper is the first to examine the simultaneous identification of vehicle and bridge parameters and road unevenness in a field test. The VBISI method is a new approach that expands the possibilities of indirect monitoring.

A limitation of this study is that it took a lot of work to find a combination of parameters to estimate the road unevenness by the studied method between the front and rear wheels. The VBISI method had to consider the effects of noise fully, and the optimization method needed to be revised. Therefore, it is conceivable to adopt a less susceptible technique to engine vibration and measurement noise in future research. Establishing a methodology to evaluate the estimated mechanical parameters of the vehicle and bridge is also essential.

A summary of the results is as follows:

1. The VBISI method can estimate the mechanical parameters of the vehicle and bridge and road unevenness from vehicle vibration and position information. The only information required for the estimation is the vehicle’s total weight and the wheelbase.
2. The Kalman filter improves the accuracy of estimating road unevenness but reduces the accuracy of estimating the mechanical parameters of the vehicle and bridge.
3. The method was validated with vehicle vibrations measured during field tests. The values estimated by the proposed method are compared to the directly measured vibrations.
4. It is not easy to estimate the natural frequencies of bridges from the bridge vibrations estimated by the proposed method. On the other hand, some of the estimated road unevenness showed similar trends.
5. To improve the accuracy of the VBISI method, the vibration preprocessing and optimization methods need to be improved. In addition, a method for evaluating the estimated mechanical parameters needs to be established.

## Figures and Tables

**Table 1:** Comparison with previous studies.

	Estimated Target				
	Road	Vehicle	Bridge	Including Bridges	Kalman Filter
McGetrick et al. [49]	○			○	
He and Yang [50]	○			○	○
Yang et al. [51]	○			○	○
Hasegawa et al. [52]	○				
Xue et al. [10] Nagayama et al. [11]	○	○			○
Keenahan et al. [53]	○	○	○	○	
VBISI method [7,8,9]	○	○	○	○	○ [7,9]

**Table 2:** The assumed mechanical parameters of vehicle and bridge.

Vehicle Parameter		Assumed		Bridge Parameter		Assumed	
$d_{1}$	Distance between wheels and center of gravity	2.18	[m]	L	Length	30.0	[m]
$d_{2}$		2.67	[m]		Number of Elements	7	
$m_{s}$	Mass	13,060	[kg]	$EI_{i}$	Bending stiffness	$5.50\times 10^{10}$	[Nm^2^]
$m_{ui}$	Mass (sprung)	$3.20\times 10^{3}$	[kg]	ρA	Mass per unit	$1.80\times 10^{4}$	[kg/m]
$c_{si}$	Damping (sprung)	$3.00\times 10^{4}$	[kg/s]	α	Rayleigh damping coefficient	0.7024	
$k_{si}$	Stiffness (sprung)	$4.00\times 10^{3}$	[N/m]	β		0.0052	
$k_{ui}$	Stiffness (unsprung)	$4.00\times 10^{5}$	[N/m]				

**Table 3:** Variance–Covariance Matrix Settings.

(*i*,*i*)	(1, 1)	(2, 2)	(3, 3)	(4, 4)	(5, 5)	(6, 6)
$Q_{ii}$	$1.35\times 10^{-4}$	$1.47\times 10^{-4}$	$1.84\times 10^{-4}$	$1.85\times 10^{-4}$	$5.11\times 10^{-4}$	$5.73\times 10^{-4}$
$R_{ii}$	$4.50\times 10^{-3}$	$5.20\times 10^{-3}$	$3.34\times 10^{-3}$	$3.34\times 10^{-3}$	$1.25\times 10^{-4}$	$1.37\times 10^{-4}$
(*i*,*i*)	(7, 7)	(8, 8)	(9, 9)	(10, 10)	(11, 11)	(12, 12)
$Q_{ii}$	$1.20\times 10^{-3}$	$1.20\times 10^{-3}$	$1.00\times 10^{-9}$	$1.00\times 10^{-9}$	*1*	*1*
$R_{ii}$	$1.74\times 10^{-4}$	$1.75\times 10^{-4}$				

**Table 4:** Variance–Covariance Matrix Settings.

KF	Noise	$d_{1}$	$c_{s1}$	$c_{s2}$	$k_{s1}$	$k_{s2}$	$m_{u1}$	$m_{u2}$	$k_{u1}$	$k_{u2}$	ρA	α	bC	$EI_{1}$	$EI_{4}$	$EI_{7}$
✕	✕	1.00	1.01	1.01	1.05	1.04	1.02	1.02	1.01	1.02	1.00	0.99	1.06	1.00	0.94	0.96
✕	○	0.00	0.20	0.13	0.07	3.02	2.32	0.37	2.69	1.19	0.33	1.73	2.01	0.62	0.36	0.02
○	✕	0.96	1.22	1.05	0.93	0.94	0.92	1.02	1.05	1.07	1.09	1.06	0.94	1.05	0.89	0.94
○	○	1.13	1.82	2.11	0.70	1.41	1.05	0.93	0.68	0.85	1.60	1.86	0.84	1.07	1.08	0.38

**Table 5:** The estimated mechanical parameters of vehicle and bridge.

Parameter		Estimated	Parameter		Estimated
$d_{1}$	[m]	2.18	$EI_{1}$	[Nm^2^]	$5.48\times 10^{10}$
$d_{2}$	[m]	2.67	$EI_{2}$	[Nm^2^]	$5.35\times 10^{10}$
$m_{u1}$	[kg]	$3.25\times 10^{2}$	$EI_{3}$	[Nm^2^]	$5.75\times 10^{10}$
$m_{u2}$	[kg]	$3.27\times 10^{2}$	$EI_{4}$	[Nm^2^]	$5.19\times 10^{10}$
$c_{s1}$	[kg/s]	$3.02\times 10^{4}$	$EI_{5}$	[Nm^2^]	$5.80\times 10^{10}$
$c_{s2}$	[kg/s]	$3.04\times 10^{4}$	$EI_{6}$	[Nm^2^]	$5.38\times 10^{10}$
$k_{s1}$	[N/m]	$4.22\times 10^{3}$	$EI_{7}$	[Nm^2^]	$5.25\times 10^{10}$
$k_{s2}$	[N/m]	$4.17\times 10^{3}$	α		0.6921
$k_{u1}$	[N/m]	$4.04\times 10^{5}$	β		0.0055
$k_{u2}$	[N/m]	$4.07\times 10^{5}$	ρA	[kg]	$1.79\times 10^{4}$

## Data Availability

Data is contained within the article.
